# The prognostic value of preoperative serum liver function-related indicators in colorectal cancer

**DOI:** 10.1097/MD.0000000000046047

**Published:** 2025-12-19

**Authors:** Lang Wang, Jinshan Liu, Can Tan, Yuhang Diao, Lijuan Wang, Youyun Shi, Xinpeng Shu, Yong Cheng

**Affiliations:** a Department of Gastrointestinal Surgery, the First Affiliated Hospital of Chongqing Medical University, Chongqing, China, 400016; b Department of General Surgery, Chongqing Hospital of Jiangsu Province Hospital, 401420, China. (The People’s Hospital of Qijiang District, Chongqing, 401420, China).

**Keywords:** alkaline phosphatase, colorectal cancer, hepatic function, lactate dehydrogenase, prognosis

## Abstract

The prognosis for patients with colorectal cancer (CRC) remains a significant clinical challenge. Routine and readily available serum biomarkers reflecting the liver function may offer crucial prognostic insights beyond tumor anatomy. The aim of this study was to analyze the impact of these preoperative liver function-related indicators on overall survival (OS) and to establish a novel, comprehensive model for accurate and individualized survival prediction. In this retrospective study, a total of 3938 patients with postoperative CRC were enrolled and randomly divided into the development group (n = 2758) and the validation group (n = 1180). Univariate analysis and Cox regression analysis were used to evaluate the prognostic factors, and finally a nomogram with predictive value was established. The predictive utility of the model was verified by validation group. The univariate and multivariate Cox regression analyses indicated age, tumor stage, tumor size, postoperative complications, preoperative alkaline phosphatase (ALP) level and lactate dehydrogenase (LDH) level were considered as independent risk factors for the prognosis of patients with CRC. Based on this result, a nomogram model was established. The model demonstrated excellent discriminative ability, with a C-index of 0.747 (95% CI: 0.723–0.773) in the development group and a C-index of 0.749 (95% CI: 0.709–0.782) in the validation group. The time-dependent ROC curves indicated good specificity and sensitivity in both cohorts. Calibration curves showed favorable consistency between the predicted and observed survival probabilities. We identified preoperative ALP and LDH levels as independent prognostic factors in CRC. The nomogram based on age, tumor stage, tumor size, postoperative complications, preoperative ALP and LDH levels could predict the postoperative survival of CRC patients, facilitating better clinical decision-making.

## 1. Introduction

Colorectal cancer (CRC) is one of the most common malignant tumors worldwide. Currently, it ranks third in morbidity and second in mortality.^[1]^ With the progress of early screening, chemoradiotherapy, immunotherapy, and targeted therapy, the 5-year survival rate of CRC has increased from 50% to 64%,^[2,3]^ but surgical treatment is still the most decisive treatment.^[4–6]^ However, there is still a lack of effective prognostic markers to predict treatment response and guide clinical decision-making.

In patients with CRC, the liver is not only the most common, but also the most prognostically associated site of metastasis.^[7–9]^ In addition, certain baseline liver function-related indicators have also been suggested to be closely related to the prognosis of patients with CRC in recent studies.^[10,11]^

In clinical practice, the routine detection of liver enzymes related indicators include glutamate aminotransferase (ALT), aspartate aminotransferase (AST), lactate dehydrogenase (LDH), alkaline phosphatase (ALP), γ-glutamyl transpeptidase (GGT), and cholinesterase (CHE). Hypoxic microenvironment and glycolysis, also known as Warburg effect, are the metabolic characteristics of malignant tumor cells, including CRC.^[12,13]^ High LDH levels were associated with worse progression-free survival and overall survival (OS).^[14,15]^ Overexpression of GGT was observed in many tumors, including CRC.^[16,17]^ Elevated GGT levels are associated with tumor invasion and metastasis and often indicate a poor prognosis. ALP is mainly derived from the liver and bone, and its elevated serum level is often associated with liver diseases, such as hepatitis, cirrhosis, and liver tumors.^[18]^ It has recently been suggested that increased ALP levels might also be associated with CRC progression and poor prognosis.^[19,20]^ Notably, considering the role of serum albumin (ALB) and bilirubin in reflecting liver function, the albumin-bilirubin (ALBI) score, initially used to assess liver function,^[21]^ has also been reported as an independent risk factor for CRC patients undergoing radical resection.^[22,23]^

Although the prognostic role of the above liver function-related markers in CRC has been widely reported, their predictive value in an integrated clinical setting remains unclear. The nomogram, serving as a robust predictive tool, provides a highly accurate and visualized means of quantifying clinical prognosis for individual patients.^[24]^ Therefore, we aimed to establish a nomogram, comprehensively considering these liver function-related markers along with clinicopathological characteristics, to predict the long-term oncology outcomes and guide individualized treatment for postoperative CRC patients.

## 2. Materials and methods

### 2.1. Recruitment of patients

A total of 3938 patients with CRC who underwent radical resection at Department of Gastrointestinal Surgery, the First Affiliated Hospital of Chongqing Medical University from January 2011 to December 2020 were included in this retrospective study. The ratio of the number of participants in the development group to the validation group was 7:3. The inclusion criteria were listed as follows: age ≥18 years old; the diagnosis of CRC was confirmed by histopathology; Eastern Cooperative Oncology Group status ≤ 2; patients had received radical surgery. Exclusion criteria were listed as follows: diagnosis of any other diseases that affect liver function; previous history of other tumors. The study was approved by the Clinical Research Ethics Committee of the First Affiliated Hospital of Chongqing Medical University (approval number: 2022-135-2), and informed consent was obtained from all participants.

### 2.2. Clinical variables extracted for analysis

The following clinical and laboratory-relevant baseline data were collected from each enrolled patient. Basic information and clinical data included: Age, sex, body mass index, tumor location, tumor size, tumor staging, smoking history, drinking history, hypertension, type 2 diabetes mellitus (T2DM). The laboratory data included: hemoglobin (Hb), platelet (PLT), total bilirubin (TBil), ALB, ALT, AST, ALP, GGT, CHE. And the ALBI score was determined using the following formulas: ALBI score = (log10 bilirubin (µmol/L) × 0.66) + (albumin (g/L) × − 0.0852). Serological indicators were tested within 1 week before surgery for routine blood examinations and biochemical tests.

### 2.3. Statistical analyses

OS was defined as the interval time between radical surgery and death from any cause or the final follow-up. Variables with missing data were excluded from the analysis. For continuous variables, the independent sample t test was used to analyze the differences between groups, and for categorical variables, the Chi-square test or Fisher exact test was used. Univariate analysis was used to verify the relationship between clinicopathological factors and survival rate. After univariate analysis, variables with *P* < .05 were included in the Cox proportional hazards analysis. To construct the nomogram model, the R software 4.4.1 was used. The performance of the newly established nomogram model was tested by development and validation group. The concordance index (C-index), area under the ROC curve (AUC) and decision-curve analysis^[25]^ was used to verify the effectiveness and practicality of the nomogram model, and the accuracy of the nomogram was further evaluated by the calibration curve.^[26,27]^ *P* < .05 signifies the significant difference, and all confidence intervals were at the level of 95% confidence.

## 3. Results

### 3.1. Clinical features of included participants

According to the inclusion and exclusion criteria, a total of 3938 patients were included in our study. The median age of the patients were 64 years (55–72), comprising 2331 (59%) males and 1607 (41%) females. At a 7:3 ratio, 2758 patients were randomly assigned to the development group and 1180 patients to the validation group. There were no significant differences in baseline clinical and laboratory data between the 2 groups. Detailed baseline characteristics are shown in Table 1.

**Table 1:** Baseline information between the development and validation cohorts.

Characteristic	Overall (3938)	Development (2758)	Validation (1180)	*P*-value
Sex				
Male	2331 (59%)	1626 (59%)	705 (60%)	.644
Female	1607 (41%)	1132 (41%)	475 (40%)	
Age, year	64 (55, 72)	64 (55, 72)	64 (55, 71)	.520
BMI, kg/m^2^	22.6 (20.5, 24.8)	22.5 (20.5, 24.8)	22.7 (20.5, 24.7)	.590
Smoking (yes/no)	1497 (38%)	1053 (38%)	444 (38%)	.743
Drinking (yes/no)	1214 (31%)	872 (32%)	342 (29%)	.101
Hypertension (yes/no)	1031 (26%)	707 (26%)	324 (27%)	.233
T2DM (yes/no)	497 (13%)	356 (13%)	141 (12%)	.407
Tumor location				
Colon	1875 (48%)	1305 (47%)	570 (48%)	.569
Rectum	2063 (52%)	1453 (53%)	610 (52%)	
Tumor stage				
I	737 (19%)	510 (18%)	227 (19%)	.950
II	1606 (41%)	1130 (41%)	476 (40%)	
III	1415 (36%)	991 (36%)	424 (36%)	
IV	180 (4.6%)	127 (4.6%)	53 (4.5%)	
Tumor size				
< 5 cm	2275 (58%)	1579 (57%)	696 (59%)	.314
≥ 5 cm	1663 (42%)	1179 (43%)	484 (41%)	
Complication (yes/no)	847 (22%)	588 (21%)	259 (22%)	.660
Hb, g/L	125 (107–139)	124 (107–139)	126 (107–139)	.633
PLT, 10^9^/L	217 (170–273)	218 (170–272)	213 (168–274)	.410
Alb, g/L	40.0 (37.0–44.0)	40.0 (37.0–44.0)	40.0 (37.0–44.0)	.481
Tbil, umol/L	10.3 (7.4– 14.3)	10.3 (7.4–14.3)	10.4 (7.4–14.3)	.902
ALT, U/L	20 (14–28)	20 (14–28)	21 (14–28)	.620
AST, U/L	21 (17–26)	21 (17–26)	21 (17–27)	.089
ALP, U/L	73 (61–87)	73 (61–86)	74 (61–87)	.289
GGT, U/L	19 (14–29)	19 (14–30)	20 (14–29)	.810
LDH, U/L	307 (161–400)	305 (161–401)	313 (163–398)	.293
CHE, U/L	6845 (5667–8086)	6870 (5652–8120)	6806 (5705–8010)	.380
ALBI	−2.77 (−3.04 to −2.47)	−2.77 (−3.04 to −2.46)	−2.78 (−3.06 to −2.47)	.438

Variables are expressed as the median (Q1, Q3), n (%).

Alb = albumin, ALBI = ALbumin-Bilirubin score, ALP = alkaline phosphatase, AST = aspartate aminotransferase, BMI = body mass index, CHE = cholinesterase, GGT = γ-glutamyl transpeptidase, Hb = hemoglobin, LDH = lactate dehydrogenase, PLT = platelet, T2DM = type 2 diabetes mellitus, TBil = total bilirubin.

### 3.2. Effect of liver indicators on the prognosis of CRC patients

Our univariate Cox regression analysis indicated that age, body mass index, tumor stage, tumor size, complication, Hb, Alb, AST, ALP, LDH, CHE, ALBI were associated with OS (*P* < .05). Of those factors incorporated into the subsequent multivariate analysis, age (HR = 1.037, 95% CI = 1.027–1.047, *P* < .001), tumor stage (HR = 3.393, 95% CI = 2.283–5.045, *P* < .001), tumor size (HR = 1.237, 95% CI = 1.003–1.527, *P* = .047), complication (HR = 1.596, 95% CI = 1.286–1.980, *P* < .001), ALP level (HR = 1.003, 95% CI = 1.001–1.006, *P* = .006) and LDH level (HR = 1.000, 95% CI = 1.000–1.001, *P* = .002) were independent risk factors for the prognosis of patients with CRC (Table 2).

**Table 2:** Univariate and multivariate analysis of overall survival.

Risk factors	Univariate analysis		Multivariate analysis	
	HR (95% CI)	*P*-value	HR (95% CI)	*P*-value
Age (year)	1.044 (1.035–1.054)	<.001*	1.037 (1.027–1.047)	<.001*
Sex (male/female)	0.902 (0.734–1.108)	.325	–	–
BMI (kg/m^2^)	0.948 (0.918–0.979)	<.001*	0.987 (0.955–1.020)	.433
Hypertension (yes/no)	0.974 (0.772–1.229)	.823	–	–
T2DM (yes/no)	1.167 (0.857–0.871)	.302	–	–
Smoking (yes/no)	0.994 (0.809–1.221)	.951	–	–
Drinking (yes/no)	1.050 (0.848–1.300)	.656	–	–
Tumor location (colon/ rectum)	1.153 (0.944–1.409)	.163	–	–
Tumor stage (IV/III/II/I)	–	<.001*	–	<.001*
I	Reference	–	Reference	–
II	1.670 (1.109–2.515)	–	1.468 (0.967–2.228)	–
III	3.815 (2.584–5.634)	–	3.393 (2.283–5.045)	–
IV	10.99 (6.905–17.48)	–	9.459 (5.892–15.18)	–
Tumor size (≥ 5cm/<5cm)	1.490 (1.219–1.821)	<.001*	1.237 (1.003–1.527)	.047*
Complication (yes/no)	1.954 (1.584–2.410)	<.001*	1.596 (1.286–1.980)	<.001*
Hb, g/L	0.993 (0.989–0.997)	<.001*	1.002 (0.997–1.007)	.430
Alb, g/L	0.955 (0.939–0.971)	<.001*	0.989 (0.967–1.012)	.348
Tbil, umol/L	1.016 (1.001–1.032)	.043*	–	–
PLT, 10^9^/L	1.000 (0.998–1.001)	.387	–	–
ALT, U/L	1.001 (0.995–1.007)	.733	–	–
AST, U/L	1.009 (1.003–1.015)	<.001*	1.005 (0.998–1.013)	.141
ALP, U/L	1.005 (1.002–1.007)	<.001*	1.003 (1.001–1.006)	.006*
GGT, U/L	1.000 (0.999–1.000)	.357	–	–
LDH, U/L	1.000 (1.000–1.001)	<.001*	1.000 (1.000–1.001)	.002*
CHE, U/L	0.999 (0.998–0.999)	<.001*	1.000 (0.999–1.000)	.226
ALBI	1.878 (1.531–2.303)	<.001*	1.216 (0.933–1.583)	.147

Alb = albumin, ALBI = albumin-bilirubin score, ALP = alkaline phosphatase, AST = aspartate aminotransferase, BMI = body mass index, BMI = body mass index, CHE = cholinesterase, CI = confidence interval, GGT = γ-glutamyl transpeptidase, Hb = hemoglobin, HR = hazard ratio, LDH = lactate dehydrogenase, PLT = platelet, T2DM = type 2 diabetes mellitus, T2DM = type 2 diabetes mellitus, TBil = total bilirubin.

* *P*-value <.05.

### 3.3. Construction and validation of model

**Figure 1.**
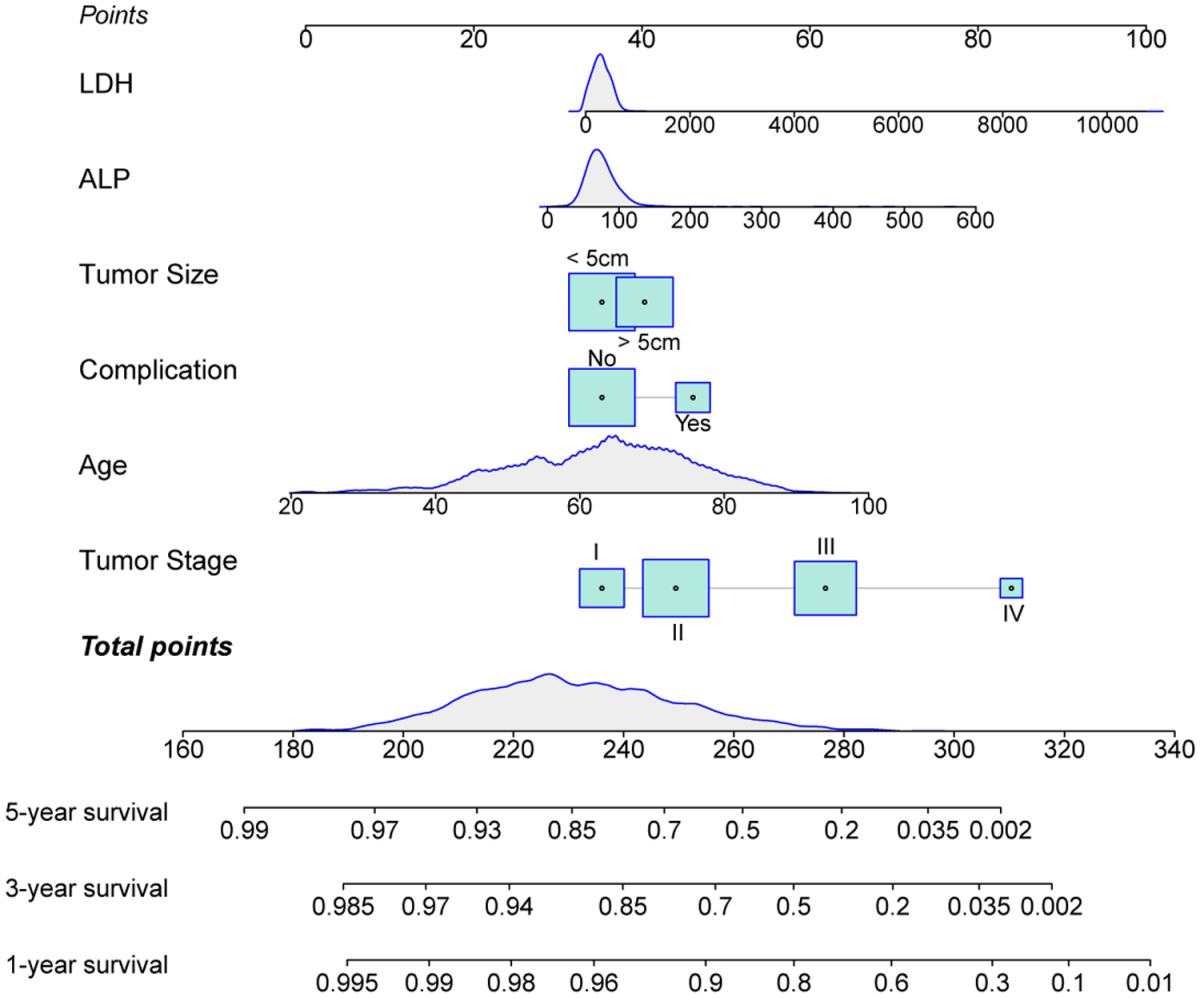
Nomogram for predicting the prognosis of patients with CRC. ALP = alkaline phosphatase, CRC = colorectal cancer, LDH = lactate dehydrogenase.

**Figure 2.**
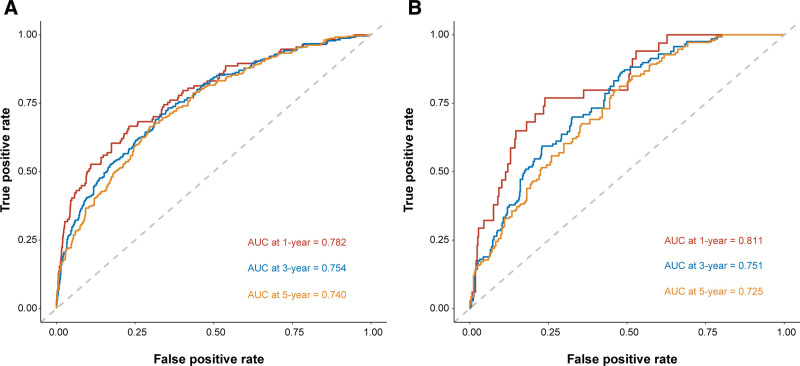
ROC curves of the nomogram. 1-, 3- and 5-year ROC curves for development group (A) and validation group (B). AUC = area under the curve, OS = overall survival, ROC = receiver operating characteristic.

**Figure 3.**
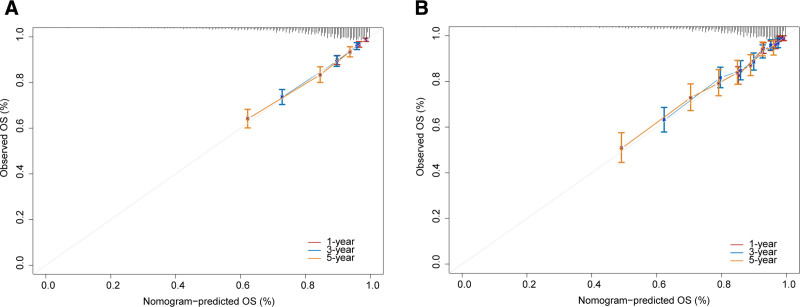
Calibration curves for the nomogram. 1-, 3- and 5-year OS calibration curves for development group (A) and validation group (B). OS = overall survival.

The nomogram was constructed based on the results of the Cox regression analysis. As shown in the Figure 1, scores were obtained according to the patient’s own age, tumor stage, tumor size, presence or absence of postoperative complications, and preoperative ALP and LDH levels, and the 5 scores were summed to yield a total score. Then, according to the total score, the 1-, 3-, and 5-year survival probabilities for CRC patients was estimated. Notably, our model demonstrated good performance with the C-index of 0.747 (95% CI: 0.723–0.773) in the development group and a C-index of 0.749 (95% CI: 0.709–0.782) in the validation group. And the ROC curves furtherly confirmed its good ability, yielding AUCs for 1-, 3-, and 5-year survival rates of 0.782, 0.754, and 0.740 in the development group and 0.811, 0.751, and 0.725 in the validation group, respectively (Fig. 2). We then plotted the nomogram calibration curve by comparing the predicted survival rate of the nomogram with the actual survival rate obtained by the Kaplan–Meier method (Fig. 3). For both the development and validation groups, the calibration curves showed no significant deviation from the reference line and did not require recalibration.

**Figure 4.**
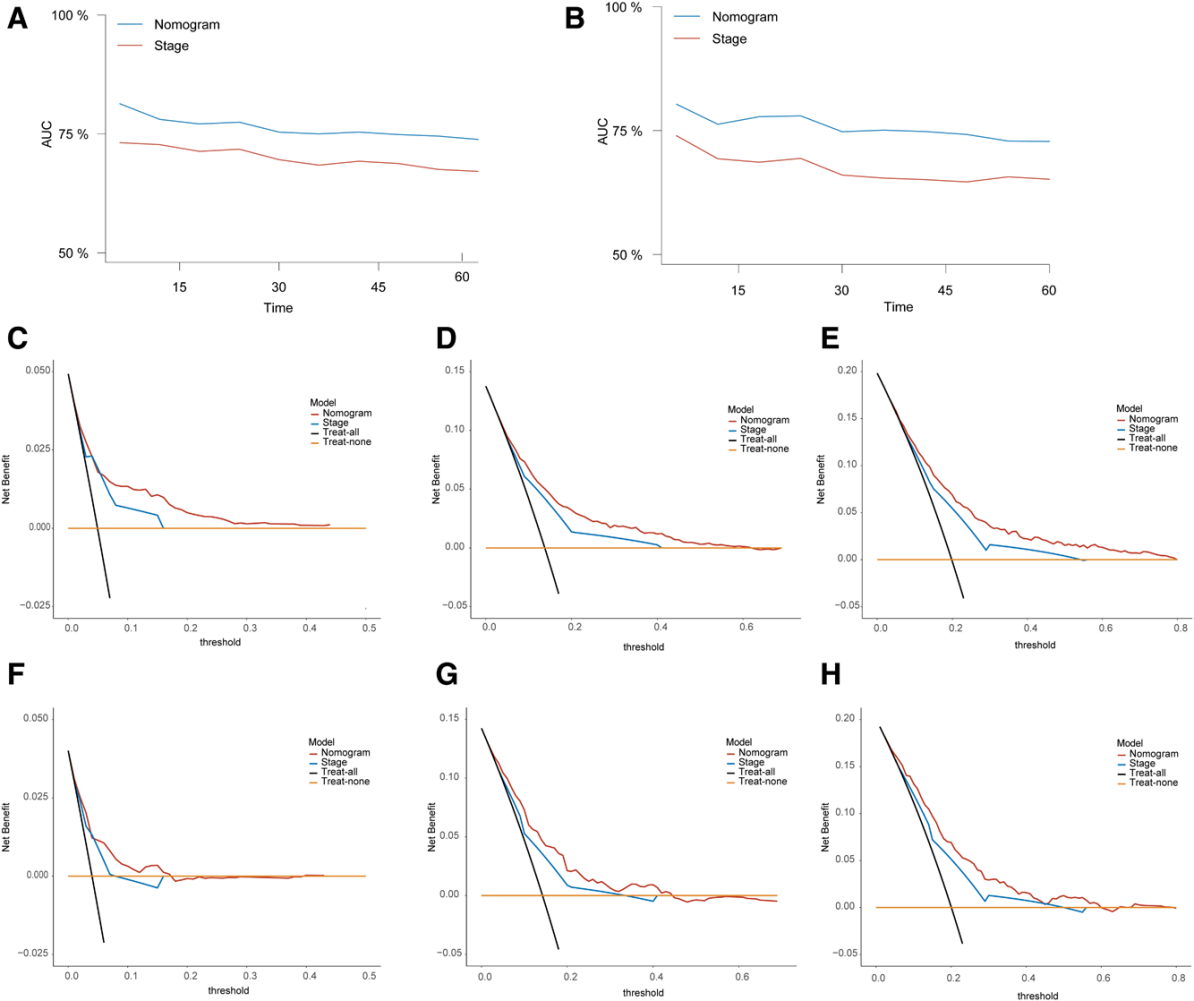
Comparison between nomogram and 8^th^ AJCC staging system. (A and B) Time-dependent ROC curves comparing the predictive ability of the nomogram and the 8^th^ AJCC staging system in the development group (A) and validation group (B). (C–H) DCA curves of the nomogram and the 8^th^ AJCC staging system for predicting 1-, 3-, and 5-year OS in the development group (C–E) and validation group (F–H). DCA = decision-curve analysis, OS = overall survival, ROC = receiver operating characteristic.

Comparing the predictive ability of the 8^th^ AJCC staging system, our nomogram demonstrated a superior performance. In the analysis, the AUC of the nomogram was consistently higher than that of the 8^th^ AJCC staging in both the development and validation cohorts (Fig. 4A and B). In addition, the decision-curve analysis showed that our nomogram offered a greater clinical net benefit within the clinically relevant range of threshold probabilities (Fig. 4C–H).

## 4. Discussion

It is well known that the prognosis of CRC is closely related to TNM stage, but there are still great differences between patients with the same stage. Therefore, it is necessary to combine new factors to assist in judging the prognosis of CRC.^[28]^ In recent years, more and more blood biomarkers have been found to be associated with the prognosis of CRC, based on which prognostic models have been established, such as tumor markers CEA and CA19-9,^[29–32]^ inflammation-related markers,^[33]^ etc. Liver function-related blood markers, including ALB, LDH, ALP, and GGT,^[17,19]^ have been confirmed to be associated with the prognosis of CRC by other studies, but their predictive value is still unclear. Here, we explored the relationship between liver function-related blood markers and the prognosis of CRC.

In this study, after COX regression analysis, we finally determined that age, tumor stage, tumor size, postoperative complications, ALP and LDH levels were associated with prognosis, and a nomogram was established based on this result. A total of 8 liver function-related markers were included in the analysis, but different from the results of some other studies, we finally found that only 2 indicators, LDH and ALP, were correlated with prognosis.

Glycolytic metabolism is one of the characteristics that distinguish tumors from normal tissues.^[34]^ Even in the presence of normal oxygen levels, tumor cells still consume glucose primarily by glycolysis.^[35]^ LDH is an enzyme that catalyzes the reversible conversion of pyruvate to lactate,^[36]^ which is released into the blood in response to cell damage or death. The up-regulation of LDH ensures efficient energy utilization by tumor cells, and the resulting acidic environment also further promotes tumor proliferation and invasion.^[37–39]^ In addition, LDH 5 was also found to be closely related to the activation of vascular endothelial growth factor pathway, thereby promoting tumor vascular proliferation.^[40]^ In CRC, elevated serum LDH level was associated with poor OS.^[41–43]^

ALP is a group of enzymes that catalyze the hydrolysis of phosphate esters to inorganic phosphates in an alkaline environment.^[44]^ Elevated ALP levels do not always indicate liver disease, but are also associated with occult cancer cell proliferation and advanced cancer states.^[18]^ It has been found to be associated with a variety of cancers, including lung cancer, pancreatic cancer, and breast cancer.^[45–48]^ In CRC, elevated ALP levels often indicated tumor progression and liver metastasis.^[19]^

Although the ALBI score has demonstrated prognostic utility in other CRC cohorts,^[22,23]^ it did not retain independent significance in our analysis. This discrepancy, in contrast to established literature, may be attributed to the diversity of the patient population and the potential selection bias inherent in retrospective study. Similarly, while other composite markers, such as the lactate dehydrogenase-to-albumin ratio (LAR), have also been reported as prognostic indicators.^[49]^ Therefore, the systematic evaluation of such composite markers will be one of our future research directions, to be further explored within large, multi-center, prospective cohorts.

Notably, our study successfully established a novel prognostic model for patients with CRC. Compared with previous studies, our work benefited from a relatively large sample size. The AUCs of 1-, 3-, and 5-year survival rates were 0.782, 0.754, and 0.740 in the development group and 0.811, 0.751, and 0.725 in the validation group, respectively, indicating the excellent application capabilities of our model. And it also demonstrated superior predictive performance compared to the traditional AJCC staging system. However, several limitations should be acknowledged. As a single center, retrospective study, our cohort inherently lacked sufficient ethnic, regional, and socioeconomic diversity. This limitation may potentially lead to selection bias and restrict the generalizability of our findings. In addition, although the internal validation indicated good model performance, the absence of external validation hinders assessment of its broader applicability. Thus, our conclusions need to be further validated in future prospective studies with more medical centers.

## 5. Conclusion

Overall, age, tumor stage, tumor size, postoperative complication, LDH, and ALP emerged as independent prognostic risk factors for CRC patients. The survival nomogram combining preoperative serum liver function indicators and clinicopathological features provides a comprehensive prediction model, which can accurately evaluate the long-term outcomes in CRC patients undergoing radical resection. Crucially, the predictive performance and clinical benefit rate of the survival nomogram were superior to that of conventional 8^th^ AJCC staging system.

## Acknowledgments

We acknowledge all the authors whose publications are referred in our article.

## Author contributions

**Conceptualization:** Yong Cheng.

**Data curation:** Yong Cheng, Lang Wang, Jinshan Liu, Can Tan, Yuhang Diao, Lijuan Wang, Youyun Shi, Xinpeng Shu.

**Formal analysis:** Lang Wang.

**Funding acquisition:** Yong Cheng.

**Supervision:** Jinshan Liu.

**Visualization:** Lang Wang, Yuhang Diao, Youyun Shi, Xinpeng Shu.

**Writing – original draft:** Lang Wang.

**Writing – review & editing:** Yong Cheng, Jinshan Liu, Can Tan, Lijuan Wang.
